# RETRACTION: Prevalence of Different Types of Wound Infection in Subjects with Parkinson's Disease and Total Joint Arthroplasty: A Meta‐Analysis

**DOI:** 10.1111/iwj.70229

**Published:** 2025-02-11

## Abstract

**RETRACTION**: WangY. , WangS. , and YangX. , “Prevalence of Different Types of Wound Infection in Subjects with Parkinson's Disease and Total Joint Arthroplasty: A Meta‐Analysis,” International Wound Journal 20, no. 7 (2023): 2780–2787, 10.1111/iwj.14154.36924416 PMC10410355

The above article, published online on 16 March 2023, in Wiley Online Library (http://onlinelibrary.wiley.com/), has been retracted by agreement between the journal Editor in Chief, Professor Keith Harding; and John Wiley & Sons Ltd. Following an investigation by the publisher, all parties have concluded that this article was accepted solely on the basis of a compromised peer review process. The editors have therefore decided to retract the article. The authors did not respond to our notice regarding the retraction.

**RETRACTION**: Y. Wang , S. Wang , and X. Yang , “Prevalence of Different Types of Wound Infection in Subjects with Parkinson's Disease and Total Joint Arthroplasty: A Meta‐Analysis,” International Wound Journal 20, no. 7 (2023): 2780–2787, 10.1111/iwj.14154.36924416 PMC10410355

The above article, published online on 16 March 2023, in Wiley Online Library (http://onlinelibrary.wiley.com/), has been retracted by agreement between the journal Editor in Chief, Professor Keith Harding; and John Wiley & Sons Ltd. Following an investigation by the publisher, all parties have concluded that this article was accepted solely on the basis of a compromised peer review process. The editors have therefore decided to retract the article. The authors did not respond to our notice regarding the retraction.

